# High contents of very long-chain polyunsaturated fatty acids in different moss species

**DOI:** 10.1007/s00299-013-1525-z

**Published:** 2013-10-30

**Authors:** Anna K. Beike, Carsten Jaeger, Felix Zink, Eva L. Decker, Ralf Reski

**Affiliations:** 1 Plant Biotechnology, Faculty of Biology, University of Freiburg, Schänzlestraße 1, 79104 Freiburg, Germany; 2 Core Facility Metabolomics, ZBSA, Center for Biological Systems Analysis, University of Freiburg, Habsburgerstraße 49, 79104 Freiburg, Germany; 3 BIOSS-Centre for Biological Signalling Studies, 79104 Freiburg, Germany; 4 FRIAS-Freiburg Institute for Advanced Studies, 79104 Freiburg, Germany

**Keywords:** *Physcomitrella patens*, Polyunsaturated fatty acids, Arachidonic acid, In vitro cultivation, Mosses, Metabolite profiling

## Abstract

*****Key message***:**

**Mosses have high contents of polyunsaturated fatty acids. Tissue-specific differences in fatty acid contents and fatty acid desaturase (FADS)-encoding gene expression exist. The arachidonic acid-synthesizing FADS operate in the ER.**

**Abstract:**

Polyunsaturated fatty acids (PUFAs) are important cellular compounds with manifold biological functions. Many PUFAs are essential for the human diet and beneficial for human health. In this study, we report on the high amounts of very long-chain (vl) PUFAs (≥C_20_) such as arachidonic acid (AA) in seven moss species. These species were established in axenic in vitro culture, as a prerequisite for comparative metabolic studies under highly standardized laboratory conditions. In the model organism *Physcomitrella* *patens*, tissue-specific differences in the fatty acid compositions between the filamentous protonema and the leafy gametophores were observed. These metabolic differences correspond with differential gene expression of fatty acid desaturase (FADS)-encoding genes in both developmental stages, as determined via microarray analyses. Depending on the developmental stage and the species, AA amounts for 6–31 %, respectively, of the total fatty acids. Subcellular localization of the corresponding FADS revealed the endoplasmic reticulum as the cellular compartment for AA synthesis. Our results show that vlPUFAs are highly abundant metabolites in mosses. Standardized cultivation techniques using photobioreactors along with the availability of the *P.* *patens* genome sequence and the high rate of homologous recombination are the basis for targeted metabolic engineering in moss. The potential of producing vlPUFAs of interest from mosses will be highlighted as a promising area in plant biotechnology.

**Electronic supplementary material:**

The online version of this article (doi:10.1007/s00299-013-1525-z) contains supplementary material, which is available to authorized users.

## Introduction

Polyunsaturated fatty acids (PUFAs) are ubiquitous metabolites with a large variety of biological functions. Their functions range from key roles in cellular signaling as precursors of hormones and phytohormones to the maintenance of membrane integrity and dynamics as major components of the biomembrane system. Many very long-chain (vl) PUFAs (≥C_20_), especially ω-3 PUFAs, are beneficial for human health as they contribute to the prevention of cardiovascular and inflammatory diseases (Calder 2004; Xue et al. 2013). Vl ω-6 PUFAs such as dihomo-γ-linolenic acid (DGLA, 20:3^Δ8,11,14^) and arachidonic acid (AA, 20:4^Δ5,8,11,14^) as well as the ω-3 vlPUFA eicosapentaenoic acid (EPA, 20:5^Δ5,8,11,14,17^) are the precursors of biologically active signaling compounds in humans, namely, eicosanoid hormones, which comprise prostaglandins, leukotrienes and thromboxanes (Samuelsson 1983; Harizi et al. 2008). Eicosanoid hormones mediate important physiological processes such as hypersensitivity reactions and inflammatory responses, but also immunity (Samuelsson 1983; Samuelsson et al. 1987; Harizi et al. 2008). Furthermore, the semi-essential fatty acid AA plays an important role in infant nutrition, as AA levels correlate with first year growth of preterm infants (Carlson et al. 1993).

Essential PUFAs for the human diet are linoleic acid (LA, 18:2^Δ9,12^), α-(ALA, 18:3^Δ9,12,15^) and γ-linolenic acid (GLA, 18:3 ^Δ6,9,12^) that need to be ingested via plant-based nutrition, while nutritional sources for AA and EPA are mainly marine fishes (Gill and Valivety 1997). However, alternative sources for AA can also be bacteria, fungi (Yuan et al. 2002), algae (Bigogno et al. 2002) and mosses (Hartmann et al. 1986; Girke et al. 1998; Kaewsuwan et al. 2006). In contrast to mosses which contain large amounts of vlPUFAs (Grimsley et al. 1981; Hartmann et al. 1986; Girke et al. 1998; Zank et al. 2002; Mikami and Hartmann 2004; Kaewsuwan et al. 2006), higher plants rarely possess these as they lack the corresponding enzymes for vlPUFA-synthesis (Gill and Valivety 1997). In the moss model organism, *Physcomitrella patens*, the genes that encode the key enzymes of AA synthesis, namely Δ6- and a Δ5-fatty acid desaturases (FADS) and a Δ5-fatty acid elongase have already been identified via targeted gene replacement and biochemical characterization (Girke et al. 1998; Zank et al. 2002; Kaewsuwan et al. 2006). Recently, also two *P.* *patens* Δ12-FADS, that are associated with linoleic acid biosynthesis, were identified and characterized by heterologous expression in the yeast *Saccharomyces cerevisiae* (Chodok et al. 2013).

The high abundance of vlPUFAs, which are uncommon in higher plants, marks clear metabolic differences between mosses and higher plants. On the one hand the use of moss genes in a transgenic approach, e.g., for the optimization of oil seed crops as an alternative to the use of genes from microalgae or fish (Jiao and Zhang 2013), forms a promising research field. On the other hand, mosses themselves provide the potential for the discovery of yet uncharacterized metabolites (Cove et al. 2006; Asakawa 2007; Xie and Lou 2009; Erxleben et al. 2012), but also for the production of metabolites in the moss bioreactor that was established for cultivation of *P.* *patens* (Decker and Reski 2008, 2012). Due to the high rate of homologous recombination, i.e., the ability to integrate homologous nucleotide sequences into the genome, metabolic engineering, but also the production of recombinant proteins, has already been realized in *P.* *patens* (Büttner-Mainik et al. 2011; Chodok et al. 2012; Parsons et al. 2012). The high rate of homologous recombination in *P.* *patens* is unique among land plants at the current state of knowledge, being comparable with the gene targeting efficiency in yeast and several times higher than in vascular plants (Strepp et al. 1998; Schaefer 2001; Hohe et al. 2004; Kamisugi et al. 2006). Beside *P.* *patens*, homologous recombination-based gene targeting is also applicable in the moss *Ceratodon purpureus* (Brücker et al. 2005) and the liverwort *Marchantia polymorpha* (Ishizaki et al. 2013), indicating that this might be a common feature among certain Bryopsida and liverworts, thus expanding the selection of species to be analyzed with regard to genetic engineering and the production of metabolites of interest.

To quantify the abundance of vlPUFAs among Bryopsida, comparative fatty acid profiles of seven moss species from different phylogenetic groups were established. The cellular compartment of AA synthesis is the endoplasmic reticulum (ER) as confirmed via green fluorescent protein (GFP)-tagging of the AA-producing FADS from *P.* *patens*. It has previously been shown that the different developmental stages of *P.* *patens* protonema and gametophores show distinct metabolic profiles for sugar derivates, amino acids and nitrogen-rich storage compounds (Erxleben et al. 2012). Here, we established comparative fatty acid profiles of protonema and gametophores to characterize tissue-specific fatty acid contents. The observed differences in the PUFA profiles of protonema and gametophores were compared with and supported by microarray-derived gene expression profiles of putative FADS-encoding genes, which for some FADS-coding genes revealed significantly higher expression levels in protonema than in gametophores.

## Materials and methods

### Plant material and growth conditions

**Fig. 1:**
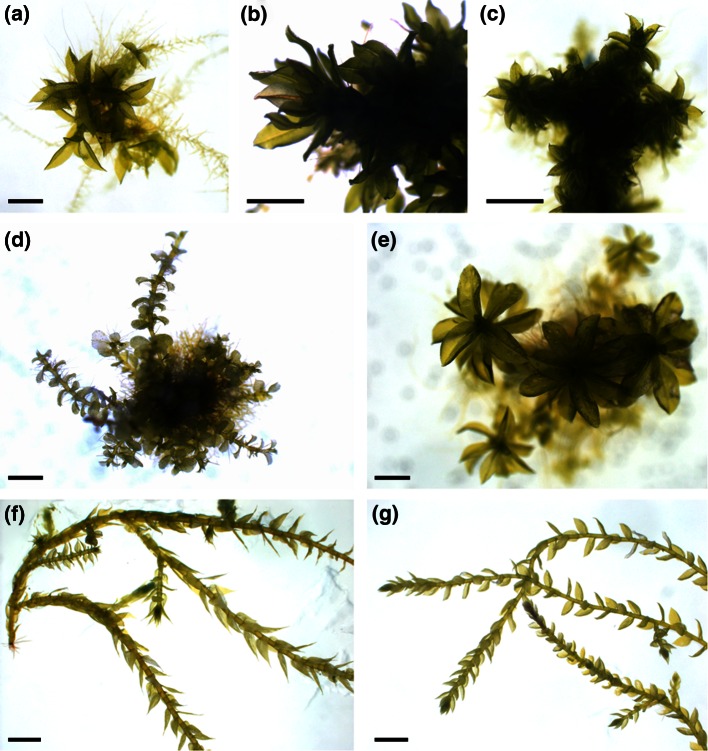
Moss species selection. Overview of the moss species grown in axenic in vitro culture and analyzed regarding their fatty acid contents. **a** *Physcomitrella* *patens*, **b** *Encalypta* *streptocarpa*, **c** *Pottia* *lanceolata*, **d** *Plagiomnium* *undulatum*, **e** *Atrichum* *undulatum*, **f** *Brachythecium rutabulum*, **g** *Rhynchostegium murale*. *Scale bar* 1 mm

With the exception of the established laboratory strain of *P.* *patens*, the moss species were collected in the field and established in axenic in vitro culture as described in Beike et al. (2010). The mosses were axenically cultivated on modified Knop medium (Reski and Abel 1985) under standardized growth conditions of 55–70 μmol m^−2^ s^−1^ light intensity and a photoperiod of 16 h light to 8 h dark at 23 ± 1 °C (Hohe et al. 2002). Gametophores were grown in Petri dishes that were enclosed with Nescofilm™ (Roth, Karlsruhe, Germany). For vegetative propagation, the gametophores were disrupted with forceps and transferred to fresh solid medium. The species collection comprises *P.* *patens, Encalypta streptocarpa*, *Pottia lanceolata*, *Plagiomnium undulatum*, *Brachythecium rutabulum*, *Rhynchostegium murale* and *Atrichum undulatum* (Fig. 1). For fatty acid and RNA extraction the plant material was harvested with forceps and transferred to liquid nitrogen until further processing. For fatty acid and RNA extraction from protonema, *P.* *patens* was grown in liquid Knop medium (Frank et al. 2005), harvested by filtering with a Büchner funnel and a vacuum pump, and immediately transferred to liquid nitrogen.

### Fatty acid extraction and GC–MS analysis

Lipid extraction from moss tissue was adapted from Welti et al. (2002). In brief, 100 mg pulverized moss tissue was transferred into 1 mL 75 °C hot isopropanol containing 0.01 % (w/v) butylated hydroxytoluene (BHT) as an antioxidant. After shaking the mixture for 15 min at 75 °C on a thermomixer (Eppendorf, Hamburg, Germany), tubes were centrifuged for 5 min (1,000×*g*, room temperature) and the supernatant was transferred to a new tube with a Pasteur pipette. The remaining pellet was re-extracted with fresh chloroform–methanol (2:1 v/v; Folch et al. 1957) containing 0.01 % BHT for 10 min at room temperature. After centrifugation, the supernatants were combined, evaporated under a stream of nitrogen and dissolved in 1.5 mL chloroform–methanol (2:1 v/v). Following addition of 0.75 volumes 1 M KCl to remove polar contaminants (Folch et al. 1957), the organic phase was isolated and evaporated under a stream of nitrogen.

Fatty acids were converted into their methyl esters by acidic esterification (Christie 1989). In brief, 1 mL 2.5 % sulfuric acid in methanol was added to the dried organic phase and esterification was carried out for 90 min at 80 °C on a thermomixer. After 5 min at room temperature, 1.5 mL 0.9 % NaCl and 1 mL hexane were added to the reaction, from which the organic phase was isolated after short mixing and centrifugation. After evaporation under nitrogen, fatty acid methyl esters were dissolved in 100 μL chloroform and transferred to GC vials. All extraction and derivatization steps were carried out in screw-cap glass tubes sealed with Teflon-coated caps. 1 μL sample aliquots were injected into an Agilent 7890A/5975C GC–MS system (Agilent, Waldbronn, Germany). A split/splitless injector was used in pulsed splitless mode at 230 °C and 9.3 psi pressure. Chromatographic separation was achieved on a 30 m × 0.25 mm × 0.25 μm HP-5MS capillary column (Agilent Technologies, Waldbronn, Germany) with helium as carrier gas at a flow rate of 1 mL/min. The temperature ramp was programmed as follows: 80 °C for 2 min, 5 °C/min increase to 325 °C, 325 °C held for 10 min. The transfer line connecting GC oven with quadrupole MS detector was heated to 260 °C. 70 eV electron impact (EI) mass spectra of eluting compounds were acquired in full-scan mode (m/z 50–500) over a total runtime of 61 min.

Peak identification was performed with the AMDIS software (Stein 1999) that integrates raw data processing (deconvolution, compound detection) and comparison of acquired mass spectra/retention times with reference libraries. To identify fatty acids, a custom reference library was created from a 37-component fatty acid methyl ester (FAME) mix (Sigma, Deisenhofen, Germany). In addition, current versions of the commercial libraries FiehnLib (Kind et al. 2009) and NIST (NIST 2008) were used. Fatty acids were considered identified when mass spectral similarity between sample and standard was 95 % or higher and retention times did not deviate more than 3 s. In cases where retention time deviation was higher, only chain length and degree of unsaturation (but not the exact structural isomer) were determined from the FAME mass spectrum where possible (Christie 1989). Such fatty acids were specified by systematic names without indication of double bond position, e.g., “hexadecadienoic acid”. For quantification, peak areas of fatty acids were determined after baseline correction and normalized to the total peak area of all fatty acids. Levels of background contamination were determined from chemical blanks, obtained by the above procedure under omission of biological material, and subtracted from sample fatty acid levels.

### Cloning of desaturase-GFP fusion constructs and protoplast transfection

For subcellular localization of the fatty acid desaturases, moss protoplasts were isolated according to Rother et al. (1994) and transiently transfected with desaturase-green fluorescent protein (GFP) fusion constructs. The fusion constructs contained the PpAct5 promoter (Weise et al. 2005) and the coding sequence (CDS) of each fatty acid desaturase, respectively (Δ5-FADS: Phypa_165175, Δ6-FADS: Phypa_164045, putative ω-3-FADS: Phypa_183309), within a GFP-reporter plasmid described before (Kiessling et al. 2004). RNA was extracted from protonema with TRIzol^®^ reagent (Invitrogen, Karlsruhe, Germany) according to the manufacturer’s protocol. Complementary DNA (cDNA) was generated with SuperScript III (Invitrogen, Karlsruhe, Germany) and PolyT-primers according to the manufacturer’s protocol. The CDS were amplified from cDNA using oligonucleotides that contained restriction enzyme binding sites (165175-GFP-SalI-for: GGTCGACATGGCGCCCCACTCTGCGGAT, 165175-GFP-Acc65I-rev: CGGTACCGCCATCGAGCCGAAACTCTGTC, 164045-GFP-Acc65I-for: GGGTACCGAAATGGTATTCGCGGGCGGTG, 164045-GFP-BglII-rev: CAGATCTACTGGTGGTAGCATGCTGCTC, 183309-GFP-XhoI-f: GCTCGAGATGGCGGCCTCTCTGTTGTCCA, 183309-GFP-BglII-r: CAGATCTGAAGGTAGGATCTGTCTGGTAG). Protoplasts were isolated and transfected as described by Hohe et al. (2004). After transfection, the protoplasts were resuspended in a regeneration medium (Rother et al. 1994) and incubated in the dark for 3–4 days before microscopic analysis.

As a control for mitochondria-specific fluorescence patterns, the protoplasts were stained with MitoTracker^®^ Orange CMTMRos (MTO, Invitrogen, Karlsruhe, Germany), a mitochondria-specific fluorescence dye. Before microscopic analysis, 1 μL MTO was added to 1 mL protoplast solution. After incubation for 10 min, the protoplasts were centrifuged at 45×*g* for 10 min. The supernatant was removed, leaving 100 μL for confocal laser scanning electron microscopy. As a control for plastid-localization, a putative ω-3-FADS predicted to be localized with 99 % probability and a confidence of 0.85 in the chloroplasts using YLoc (LowRes Plants) (Briesemeister et al. 2010) was tagged with GFP.

### Confocal laser scanning electron microscopy

Confocal microscopy was done with the Zeiss LSM 510 with inverted microscope Axiovert 200 at the Life Imaging Center (LIC, University of Freiburg). The LD LCI Plan-Apochromat 25x/0.8 DIC ImmKorr water immersion objective was used to search for transformed protoplasts, while the C-Apochromat 63x/1,2 W VIS-IRKorr water immersions objective was used to take images. For the detection of GFP and chlorophyll autofluorescence, the sample was excited with an Argon laser at 488 nm. For MTO detection a helium-neon laser at 543 nm was used. Fluorescence signals are false-colored in green (GFP), orange (MTO) and red (chlorophyll), respectively. Three-dimensional reconstruction was performed via z-stacking with the Imaris v3.1 software (Bitplane).

### Analysis of gene expression

Gene expression analyses of protonema and gametophores were performed using a Combimatrix 90 K microarray (Combimatrix Corp., Mukilteo/WA, USA) based on the v1.2 gene models of *P.* *patens* (Rensing et al. 2008) as described in Wolf et al. (2010). RNA extraction, sample preparation and computational data analysis were done as described previously (Richardt et al. 2010; Wolf et al. 2010). The microarray experiments were performed in three biological replicates. Statistical data analyses were done with the Expressionist Analyst 7.5 software (www.genedata.com, Genedata, Basel, Switzerland). The putative FADS-coding genes were selected based on the KEGG pathway database (Kanehisa and Goto 2000; Kanehisa et al. 2012) using the pathway map “Biosynthesis of unsaturated fatty acids” for *P.* *patens* (ppp01040).

### Statistical analysis

To test for significant differences between the fatty acid contents of protonema and gametophores, an unpaired *t-*test was performed with the GraphPad software (http://www.graphpad.com). Averages and standard deviations were calculated with Microsoft Excel.

## Results

### Mosses contain high amounts of vlPUFAs

**Fig. 2:**
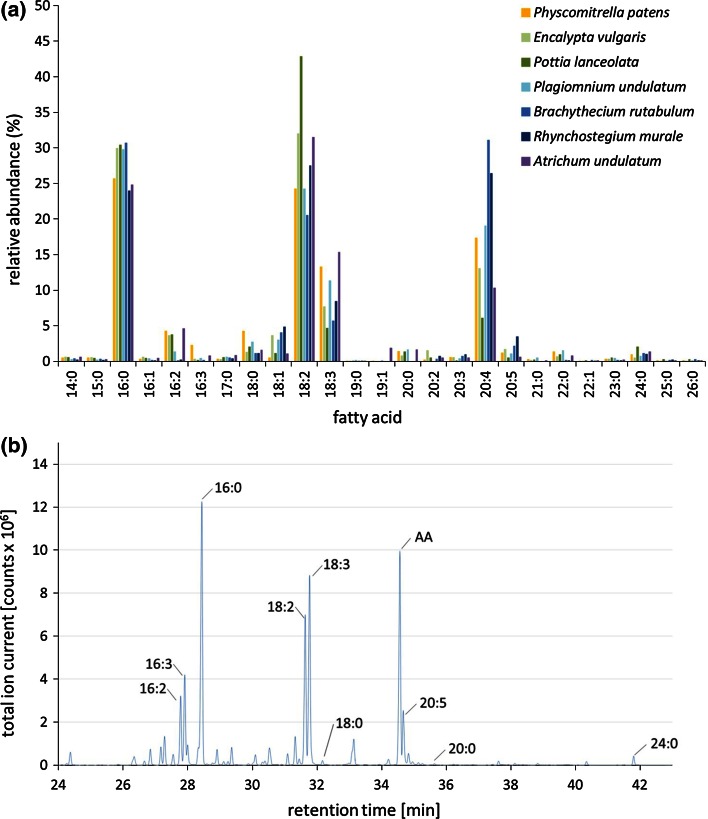
Comparative fatty acid profiles from different mosses. **a** Fatty acid profiles were established from different moss species (gametophores) that had been cultivated in vitro under axenic conditions. The *x*-axis depicts the fatty acids written in lipid numbers C:D, where C represents the number of carbon atoms and D the number of double bonds of the fatty acid. The *y*-axis depicts the relative amount of the fatty acid as a percentage of the total fatty acid content. **b** Sample GC–MS chromatogram for *P. patens*, with important fatty acid peaks indicated

The species collection comprised *P.* *patens* (Funariaceae)*, E. streptocarpa* (Encalyptaceae), *P. lanceolata* (Pottiaceae), *P. undulatum* (Mniaceae), *B. rutabulum* and *R. murale* (Brachytheciaceae), and *A. undulatum* (Polytrichaceae) (Fig. 1). These axenically cultivated moss species contained considerable amounts of vlPUFAs (>C_18_) like AA (20:4), but also smaller amounts of saturated very long-chain fatty acids such as tetra- (24:0), penta- (25:0) and hexacosanoic acid (26:0) (Fig. 2; Table S1). The predominant peak among vlPUFAs was AA in all analyzed species (Fig. 2). In *P.* *patens*, AA reached a level of 18.7 % on average in gametophores and 15.9 % in protonema (Table 1). Regarding the other species, AA contents ranged from 6 to 31 % of total fatty acids (Fig. 2). While *P. lanceolata* and *A. undulatum* had AA contents of only around 6–10 %, *B. rutabulum* and *R. murale* reached AA levels of up to 31 % of the total fatty acids (Fig. 2).

**Table 1:** Highly abundant fatty acids in *Physcomitrella patens*

Fatty acid (C:D, common name)	Gametophores (%) (±SD)	Protonema (%) (±SD)
16:0, Palmitic acid	25.3 (±0.4)	26.5 (±0.04)*
16:2, Hexadecadienoic acid	4.1 (±0.1)	6.2 (±0.5)*
16:3, Hexadecatrienoic acid	3.3 (±0.9)	6.2 (±0.3)*
18:2, Linoleic acid	19.3 (±4.3)	5.8 (±5.3)
18:3, Linolenic acid	14.8 (±1.3)	24.0 (±1.4)*
20:4, Arachidonic acid	18.7 (±1.2)	15.9 (±1.4)
20:5, Eicosapentaenoic acid	1.5 (±0.3)	6.8 (±0.5)*

This table provides an overview of the most abundant fatty acids in *P. patens*. The selection comprises fatty acids with a relative percentage of all fatty acids that was above 5 % in at least one of the analyzed tissues. Gametophores as well as protonema contain high amounts of palmitic acid, followed by linolenic acid in protonema, and linoleic acid in gametophores. Significantly higher levels of a certain fatty acid in one of the two tissues in comparison to the other one are marked with *(*p*-value < 0.05, unpaired *t*-test)

### Tissue-specific fatty acid contents correspond with differential gene expression

**Fig. 3:**
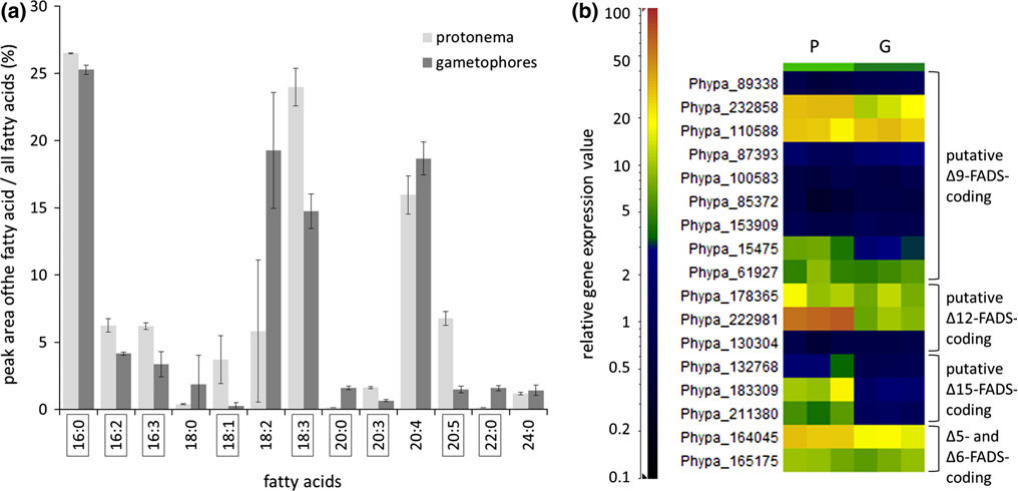
Comparative fatty acid and gene expression profiles from protonema and gametophores of *P. patens*. **a** Fatty acid profiles were established from protonema and gametophores of *P.* *patens*. The means of fatty acids with abundance higher than 1 % of all fatty acids are depicted in the *bar chart*. The *error bars* show the standard deviation. Fatty acids with significantly different abundance between both developmental stages are highlighted in *boxes* (unpaired *t*-test, *p*-value < 0.05). **b** Heat map of the relative gene expression values of putative fatty acid desaturase (FADS)-coding genes in *P.* *patens* protonema (*P*) and gametophores (*G*) represented in three biological replicates, respectively

According to our analyses *P.* *patens* contains 17.3 % AA in gametophores and protonema on average (Table 1). Further abundant fatty acids (>5 % of total fatty acids) are palmitic acid with an average of 25.9 % in gametophores and protonema, hexadecadienoic acid (16:2) with an average of 5.2 % in both developmental stages, hexadecatrienoic acid (16:3) with 6.2 % in protonema and only 3.3 % in gametophores, LA (18:2) with an average of 12.5 % in gametophores and protonema, linolenic acid (18:3) with an average of 19.4 %, and EPA with 6.8 % in protonema (Table 1). The comparative fatty acid profiles revealed significant differences in the abundance of some fatty acids in the two developmental stages (Table 1; Fig. 3a). While the saturated fatty acids arachidic acid (20:0) and behenic acid (22:0) had a significantly higher relative abundance in gametophores, the (poly-)unsaturated fatty acids hexadecadienoic acid, hexadecatrienoic acid, oleic acid, linolenic acid, dihomo-γ-linolenic acid (DGLA) and EPA had a significantly higher abundance in the juvenile protonema stage (Fig. 3a).

Corresponding to the higher relative levels of PUFAs in protonema than in gametophores, putative Δ9-, Δ12-, and Δ15-fatty acid desaturase (FADS)-encoding genes also showed a higher level of relative gene expression in protonema than in gametophores (Fig. 3b). Three of these genes (Phypa_22981, Phypa_183309, Phypa_211380) were significantly higher expressed in protonema than in gametophores (Benjamini–Hochberg-corrected *p*-value < 0.05) (Benjamini and Hochberg 1995). One putative Δ12-FADS-encoding gene (Phypa_22981) was 7.39-fold higher expressed in protonema than in gametophores, while two putative Δ15-FADS-coding genes were 7.09-fold (Phypa_183309) and 4.70-fold (Phypa_211380) higher expressed in protonema than in gametophores (Table S2). In accordance to the similar AA contents in gametophores and protonema (Fig. 3a), the AA-producing Δ5- and Δ6-FADS-encoding genes showed no significantly deviating gene expression levels in the two developmental stages (Fig. 3b).

### Arachidonic acid is produced in the endoplasmic reticulum

**Fig. 4:**
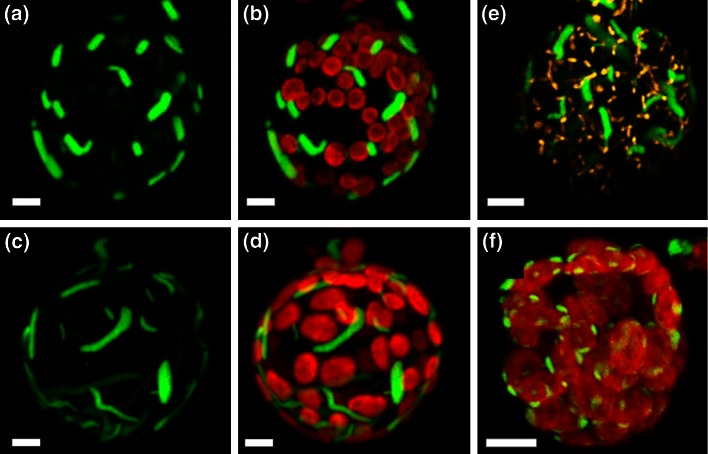
Subcellular localization of the Δ6- and Δ5-fatty acid desaturase*. Physcomitrella patens* protoplasts were transformed with a plasmid including the CDS of fatty acid desaturase-coding genes and the coding sequence for green fluorescent protein (GFP). **a** The green fluorescence of the GFP tagged to the Δ5-fatty acid desaturase was visible in the endoplasmic reticulum, while **b** chlorophyll autofluorescence of the chloroplasts was localized at distinct positions. **c** The green fluorescence of the GFP tagged to the Δ6-fatty acid desaturase was also visible in the ER, whereas **d** chlorophyll autofluorescence of the chloroplasts was localized at distinct positions. **e** Control staining with MitoTracker^®^ Orange CMTMRos (Invitrogen, Karlsruhe, Germany). **f** Subcellular localization of plastid-localized putative ω-3 fatty acid desaturase. *Scale bar* 5 μm

To determine the cellular compartment of AA synthesis, the AA-producing Δ5- and Δ6-FADS (Girke et al. 1998; Kaewsuwan et al. 2006) were tagged with green fluorescent protein (GFP). The Δ5-FADS:GFP showed GFP-fluorescence in 5 to 10 μm long and 2 μm thick accumulations along with a more reticular weaker fluorescence pattern surrounding the nucleus (Fig. 4a). This specific fluorescence pattern was distinct from chlorophyll autofluorescence (Fig. 4b). The Δ6-FADS:GFP showed comparable fluorescence patterns with accumulations and reticular structures (Fig. 4c), but without co-localization with the fluorescence of chlorophyll (Fig. 4d). We conclude that both enzymes are localized in the ER.

The control for localization in mitochondria using MitoTracker^®^ Orange CMTMRos (MTO, Invitrogen, Karlsruhe, Germany) showed mitochondria-specific fluorescence patterns distinct from the fluorescence patterns of the two AA-producing FADS:GFP (Fig. 4e). The putative ω-3-FADS is localized in the chloroplasts, showing co-localization with the fluorescence of the chlorophyll (Fig. 4f), but distinct from the fluorescence patterns of the two Δ6-FADS- and Δ5-FADS:GFP.

## Discussion

In this work, we describe seven moss species as rich sources for very long-chain PUFAs. The comparative fatty acid profiles were established from plants grown in axenic in vitro culture, a technique that we regard as a prerequisite for metabolic studies under standardized conditions. All analyzed mosses contained considerable amounts of arachidonic acid (AA, 20:4^Δ5,8,11,14^), a vlPUFA that is usually found in algae, fish and mammals. According to our analyses, AA is produced in the endoplasmic reticulum (ER) in *P.* *patens*. Beside AA smaller amounts of EPA and saturated very long-chain fatty acids (C_22–26_) were determined in all analyzed mosses. The high content of vlPUFAs in mosses highlights their potential for biotechnological application. Especially ω-3 PUFAs such as eicosapentaenoic acid (EPA, 20:5^Δ5,8,11,14,17^) and docosahexaenoic acid (DHA, 22:6^Δ4,7,10,13,16,19^) are of importance for human nutrition and need to be produced in larger amounts, as limited natural sources basically comprise algae and marine fish (Chodok et al. 2012; Xue et al. 2013). Artificial production of EPA is already achieved with metabolic engineering of the yeast *Yarrowia lipolytica* (Xue et al. 2013). However, well-directed modifications of metabolic pathways are also possible in *P.* *patens* due to its well-annotated genome sequence (Zimmer et al. 2013) and the high rate of homologous recombination in mitotic cells that facilitates the generation of genetically modified strains. This technique enables the production of vlPUFAs of interest via metabolic engineering (Kaewsuwan et al. 2010; Chodok et al. 2012). On the other hand, transgenic engineering of crops, e.g., oil seed crops using moss genes, as recently reviewed regarding genes from microalgae or fish (Jiao and Zhang 2013) is also a promising research area.

The model organism *P.* *patens* has already been established as a production platform for recombinant proteins and biopharmaceuticals using highly standardized in vitro cultivation techniques in photobioreactors (Decker and Reski 2012). However, the opportunity of metabolic engineering along with cultivation under highly standardized conditions represents one step further towards the biotechnological use of mosses as PUFA sources under good manufacturing practice (GMP) conditions. Recently, the C_22_-PUFAs adrenic acid (ADA, 22:4^Δ7,10,13,16^) and the DHA-precursor ω-3 docosapentaenoic acid (DPA, 22:5 ^Δ7,10,13,16,19^) were produced in *P.* *patens* by heterologous expression of a Δ5-elongase from a marine alga (Kaewsuwan et al. 2010; Chodok et al. 2012). Considering the biotechnological techniques available, an increased production of the ω-3 fatty acids EPA or DHA might also be possible in *P. patens* and other mosses.

However, it should be taken into account that fatty acid profiles from different developmental stages showed remarkable differences with regard to PUFA contents in *P.* *patens*. These findings are in accordance with the previously reported distinct metabolic profiles of protonema and gametophores regarding saccharides, sugar derivates, amino acids and nitrogen-rich storage compounds (Erxleben et al. 2012). According to our analyses, the relative amounts of PUFAs were higher in protonema than in gametophores, a finding that is supported by the significantly increased expression of putative fatty acid desaturase (FADS)-encoding genes in protonema when compared with the gene expression level in gametophores.

The biological meaning of the higher PUFA levels in protonema in comparison to gametophores remains a question for further research. It is known that PUFAs including AA form the precursors of signaling molecules, which are collectively named oxylipins (Andreou et al. 2009; Stumpe et al. 2010; Scholz et al. 2012). Oxylipins are produced by lipid peroxidation based on the enzymatic activity of lipoxygenases and occur in bacteria, algae, plants, fungi and animals (Andreou et al. 2009). In *P.* *patens* oxylipins can be produced from C_20_ and C_18_ fatty acids, while in seed plants oxylipins are produced from C_18_ fatty acids only (Wichard et al. 2005; Anterola et al. 2009). As recently shown for the moss *Dicranum* *scoparium* oxylipins possess anti-feeding activity against slugs and contribute to biochemical defense mechanisms (Rempt and Pohnert 2010). In *P.* *patens*, cyclopentenone–oxylipins, which are precursors of the phytohormone jasmonic acid in vascular plants, accumulate during pathogen attack by the fungus *Botrytis cinerea* (Ponce de León et al. 2012). Furthermore, cyclopentenone–oxylipins contribute to fertility and sporogenesis of *P. patens* (Stumpe et al. 2010). However, the lipid-derived phytohormone jasmonic acid itself has not been detected in this moss so far (Stumpe et al. 2010; Ponce de León et al. 2012). Considering this, clear differences not only in the lipid metabolism, but also in lipid-derived signaling exist between mosses and higher plants. The high contents of vlPUFAs may represent a key physiological characteristic that contributes to the considerable biotic and abiotic stress tolerance of mosses.

## Electronic supplementary material

Below is the link to the electronic supplementary material.

Supplementary material 1 (XLSX 17 kb)

Supplementary material 2 (XLSX 10 kb)

## References

[CR1] Andreou A, Brodhun F, Feussner I (2009). Biosynthesis of oxylipins in non-mammals. Prog Lipid Res.

[CR2] Anterola A, Göbel C, Hornung E, Sellhorn G, Feussner I, Grimes H (2009). *Physcomitrella patens* has lipoxygenases for both eicosanoid and octadecanoid pathways. Phytochemistry.

[CR3] Asakawa Y (2007). Biologically active compounds from bryophytes. Pure Appl Chem.

[CR4] Beike AK, Horst NA, Rensing SA (2010) Technical notes: axenic bryophyte in vitro cultivation. J Endocytobiosis Cell Res:102–108

[CR5] Benjamini Y, Hochberg Y (1995). Controlling the false discovery rate: a practical and powerful approach to multiple testing. J R Stat Soc B.

[CR6] Bigogno C, Khozin-Goldberg I, Boussiba S, Vonshak A, Cohen Z (2002). Lipid and fatty acid composition of the green oleaginous alga *Parietochloris incisa*, the richest plant source of arachidonic acid. Phytochemistry.

[CR7] Briesemeister S, Rahnenführer J, Kohlbacher O (2010). YLoc––an interpretable web server for predicting subcellular localization. Nucl Acids Res.

[CR8] Brücker G, Mittmann F, Hartmann E, Lamparter T (2005). Targeted site-directed mutagenesis of a heme oxygenase locus by gene replacement in the moss *Ceratodon purpureus*. Planta.

[CR9] Büttner-Mainik A, Parsons J, Jérome H, Hartmann A, Lamer S, Schaaf A, Schlosser A, Zipfel PF, Reski R, Decker EL (2011). Production of biologically active recombinant human factor H in *Physcomitrella*. Plant Biotechnol J.

[CR10] Calder PC (2004). n-3 Fatty acids and cardiovascular disease: evidence explained and mechanisms explored. Clin Sci.

[CR11] Carlson SE, Werkman SH, Peeples JM, Cooke RJ, Tolley EA (1993). Arachidonic acid status correlates with first year growth in preterm infants. Proc Natl Acad Sci USA.

[CR12] Chodok P, Cove DJ, Quatrano RS, Kanjana-Opas A, Kaewsuwan S (2012). Metabolic engineering and oil supplementation of *Physcomitrella patens* for activation of C_22_ polyunsaturated fatty acid production. J Am Oil Chem Soc.

[CR13] Chodok P, Eiamsa-Ard P, Cove DJ, Quatrano RS, Kaewsuwan S (2013). Identification and functional characterization of two Δ(12)-fatty acid desaturases associated with essential linoleic acid biosynthesis in *Physcomitrella patens*. J Ind Microbiol Biotechnol.

[CR14] Christie WW (1989). Gas chromatography and lipids.

[CR15] Cove D, Bezanilla M, Harries P, Quatrano R (2006). Mosses as model systems for the study of metabolism and development. Ann Rev Plant Biol.

[CR16] Decker EL, Reski R (2008). Current achievements in the production of complex biopharmaceuticals with moss bioreactors. Bioproc Biosys Eng.

[CR17] Decker EL, Reski R (2012). Glycoprotein production in moss bioreactors. Plant Cell Rep.

[CR18] Erxleben A, Gessler A, Vervliet-Scheebaum M, Reski R (2012). Metabolite profiling of the moss *Physcomitrella patens* reveals evolutionary conservation of osmo-protective substances. Plant Cell Rep.

[CR19] Folch J, Lees M, Stanley GHS (1957). A simple method for the isolation and purification of total lipids from animal tissues. J Biol Chem.

[CR20] Frank W, Decker EL, Reski R (2005). Molecular tools to study *Physcomitrella patens*. Plant Biol.

[CR21] Gill I, Valivety R (1997). Polyunsaturated fatty acids, part 1: occurrence, biological activities and applications. Tren Biotechnol.

[CR22] Girke T, Schmidt H, Zähringer U, Reski R, Heinz E (1998). Identification of a novel delta 6 acyl-group desaturase by targeted gene disruption in *Physcomitrella patens*. Plant J.

[CR23] Grimsley NH, Grimsley JM, Hartmann E (1981). Fatty acid composition of mutants of the moss *Physcomitrella patens*. Phytochemistry.

[CR24] Harizi H, Corcuff JB, Gualde N (2008). Arachidonic-acid-derived eicosanoids: roles in biology and immunopathology. Tren Mol Med.

[CR25] Hartmann E, Beutelmann P, Vandekerkhove O, Euler R, Kohn G (1986). Moss cell cultures as sources of arachidonic and eicosapentaenoic acids. FEBS Lett.

[CR26] Hohe A, Rensing SA, Mildner M, Lang D, Reski R (2002). Day length and temperature strongly influence sexual reproduction and expression of a novel MADS-box gene in the moss *Physcomitrella patens*. Plant Biol.

[CR27] Hohe A, Egener T, Lucht J, Holtorf H, Reinhard C, Schween G, Reski R (2004). An improved and highly standardised transformation procedure allows efficient production of single and multiple targeted gene knockouts in a moss *Physcomitrella patens*. Curr Gen.

[CR28] Ishizaki K, Johzuka-Hisatomi Y, Ishida S, Iida S, Kohchi T (2013). Homologous recombination-mediated gene targeting in the liverwort *Marchantia polymorpha* L. Sci Rep.

[CR29] Jiao J, Zhang Y (2013). Transgenic biosynthesis of polyunsaturated fatty acids: a sustainable biochemical engineering approach for making essential fatty acids in plants and animals. Chem Rev.

[CR30] Kaewsuwan S, Cahoon EB, Perroud PF, Wiwat C, Panvisavas N, Quatrano RS, Cove DJ, Bunyapraphatsara N (2006). Identification and functional characterization of the moss *Physcomitrella patens* delta5-desaturase gene involved in arachidonic and eicosapentaenoic acid biosynthesis. J Biol Chem.

[CR31] Kaewsuwan S, Bunyapraphatsara N, Cove DJ, Quatrano RS, Chodok P (2010). High level production of adrenic acid in *Physcomitrella patens* using the algae *Pavlova sp*. Delta(5)-elongase gene. Biores Technol.

[CR32] Kamisugi Y, Schlink K, Rensing SA, Schween G, von Stackelberg M, Cuming AC, Reski R, Cove DJ (2006). The mechanism of gene targeting in *Physcomitrella patens*: homologous recombination, concatenation and multiple integration. Nucl Acids Res.

[CR33] Kanehisa M, Goto S (2000). KEGG: Kyoto encyclopedia of genes and genomes. Nucl Acids Res.

[CR34] Kanehisa M, Goto S, Sato Y, Furumichi M, Tanabe M (2012). KEGG for integration and interpretation of large-scale molecular data sets. Nucl Acids Res.

[CR35] Kiessling J, Martin A, Gremillon L, Rensing SA, Nick P, Sarnighausen E, Decker EL, Reski R (2004). Dual targeting of plastid division protein FtsZ to chloroplasts and the cytoplasm. EMBO Rep.

[CR36] Kind T, Wohlgemuth G, Lee do Y, Lu Y, Palazoglu M, Shahbaz S, Fiehn O (2009). FiehnLib: mass spectral and retention index libraries for metabolomics based on quadrupole and time-of-flight gas chromatography/mass spectrometry. Anal Chem.

[CR37] Mikami K, Hartmann E (2004) Lipid metabolism in mosses. In: Wood, AJ, Oliver MJ, Cove DJ (eds.): New Frontiers in bryology: physiology, molecular biology and functional genomics

[CR38] Parsons J, Altmann F, Arrenberg CK, Koprivova A, Beike AK, Stemmer C, Gorr G, Reski R, Decker EL (2012). Moss-based production of asialo-erythropoietin devoid of Lewis A and other plant-typical carbohydrate determinants. Plant Biotechnol J.

[CR39] Ponce De León I, Schmelz EA, Gaggero C, Castro A, Álvarez A, Montesano M (2012). *Physcomitrella patens* activates reinforcement of the cell wall, programmed cell death and accumulation of evolutionary conserved defence signals, such as salicylic acid and 12-oxo-phytodienoic acid, but not jasmonic acid, upon *Botrytis cinerea* infection. Mol Plant Pathol.

[CR40] Rempt M, Pohnert G (2010). Novel acetylenic oxylipins from the moss *Dicranum**scoparium* with anti-feeding activity against herbivorous slugs. Angew Chem Int Ed.

[CR41] Rensing SA, Lang D, Zimmer AD, Terry A, Salamov A, Shapiro H, Nishiyama T, Perroud PF, Lindquist EA, Kamisugi Y, Tanahashi T, Sakakibara K, Fujita T, Oishi K, Shin-I T, Kuroki Y, Toyoda A, Suzuki Y, Hashimoto S, Yamaguchi K, Sugano S, Kohara Y, Fujiyama A, Anterola A, Aoki S, Ashton N, Barbazuk WB, Barker E, Bennetzen JL, Blankenship R, Cho SH, Dutcher SK, Estelle M, Fawcett JA, Gundlach H, Hanada K, Heyl A, Hicks KA, Hughes J, Lohr M, Mayer K, Melkozernov A, Murata T, Nelson DR, Pils B, Prigge M, Reiss B, Renner T, Rombauts S, Rushton PJ, Sanderfoot A, Schween G, Shiu SH, Stueber K, Theodoulou FL, Tu H, Van de Peer Y, Verrier PJ, Waters E, Wood A, Yang L, Cove D, Cuming AC, Hasebe M, Lucas S, Mishler BD, Reski R, Grigoriev IV, Quatrano RS, Boore JL (2008). The *Physcomitrella* genome reveals evolutionary insights into the conquest of land by plants. Science.

[CR42] Reski R, Abel WO (1985). Induction of budding on chloronemata and caulonemata of the moss, *Physcomitrella patens*, using isopentenyladenine. Planta.

[CR43] Richardt S, Timmerhaus G, Lang D, Qudeimat E, Corrêa LG, Reski R, Rensing SA, Frank W (2010). Microarray analysis of the moss *Physcomitrella patens* reveals evolutionarily conserved transcriptional regulation of salt stress and abscisic acid signalling. Plant Mol Biol.

[CR44] Rother S, Hadeler B, Orsini JM, Abel WO, Reski R (1994). Fate of a mutant macro-chloroplast in somatic hybrids. J Plant Physiol.

[CR45] Samuelsson B (1983). Leukotrienes: mediators of immediate hypersensitivity reactions and inflammation. Science.

[CR46] Samuelsson B, Dahlén SE, Lindgren JA, Rouzer CA, Serhan CN (1987). Leukotrienes and lipoxins: structures, biosynthesis, and biological effects. Science.

[CR47] Schaefer DG (2001). Gene targeting in *Physcomitrella patens*. Curr Opin Plant Biol.

[CR48] Scholz J, Brodhun F, Hornung E, Herrfurth C, Stumpe M, Beike AK, Faltin B, Frank W, Reski R, Feussner I (2012). Biosynthesis of allene oxides in *Physcomitrella patens*. BMC Plant Biol.

[CR49] Stein E (1999). An integrated method for spectrum extraction and compound identification from gas chromatography/mass spectrometry data. J Am Soc Mass Spectrom.

[CR50] Strepp R, Scholz S, Kruse S, Speth V, Reski R (1998). Plant nuclear gene knockout reveals a role in plastid division for the homolog of the bacterial cell division protein FtsZ, an ancestral tubulin. Proc Natl Acad Sci USA.

[CR51] Stumpe M, Göbel C, Faltin B, Beike AK, Hause B, Himmelsbach K, Bode J, Kramell R, Wasternack C, Frank W, Reski R, Feussner I (2010). The moss *Physcomitrella patens* contains cyclopentenones but no jasmonates: mutations in allene oxide cyclase lead to reduced fertility and altered sporophyte morphology. New Phytol.

[CR52] Weise A, Rodriguez-Franco M, Timm B, Hermann M, Link S, Jost W, Gorr G (2005). Use of *Physcomitrella patens* actin 5′ regions for high transgene expression: importance of 5′ introns. Appl Microbiol Biotechnol.

[CR53] Welti R, Li W, Li M, Sang Y, Biesiada H, Zhou H, Rajashekar CB, Williams TD, Wang X (2002). Profiling membrane lipids in plant stress responses. J Biol Chem.

[CR54] Wichard T, Göbel C, Feussner I, Pohnert G (2005). Unprecedented lipoxygenase/hydroperoxide lyase pathways in the moss *Physcomitrella patens*. Angew Chem Int Ed.

[CR55] Wolf L, Rizzini L, Stracke R, Ulm R, Rensing SA (2010). The molecular and physiological responses of *Physcomitrella patens* to ultraviolet-B radiation. Plant Physiol.

[CR56] Xie C-F, Lou H-X (2009). Secondary metabolites in bryophytes: an ecological aspect. Chem Biodivers.

[CR57] Xue Z, Sharpe PL, Hong SP, Yadav NS, Xie D, Short DR, Damude HG, Rupert RA, Seip JE, Wang J, Pollak DW, Bostick MW, Bosak MD, Macool DJ, Hollerbach DH, Zhang H, Arcilla DM, Bledsoe SA, Croker K, McCord EF, Tyreus BD, Jackson EN, Zhu Q (2013). Production of omega-3 eicosapentaenoic acid by metabolic engineering of *Yarrowia lipolytica*. Nat Biotechnol.

[CR58] Yuan C, Wang J, Shang Y, Gong G, Yao J, Yu Z (2002). Production of arachidonic acid by *Mortierella alpina* I_49_–N_18_. Food Technol Biotechnol.

[CR59] Zank TK, Zähringer U, Beckmann C, Pohnert G, Boland W, Holtorf H, Reski R, Lerchl J, Heinz E (2002). Cloning and functional characterization of an enzyme involved in the elongation of Delta 6- polyunsaturated fatty acids from the moss *Physcomitrella patens*. Plant J.

[CR60] Zimmer AD, Lang D, Buchta K, Rombauts S, Nishiyama T, Hasebe M, van de Peer Y, Rensing SA, Reski R (2013). Reannotation and extended community resources of the non-seed plant *Physcomitrella**patens* provide insights into the evolution of plant gene structures and functions. BMC Gen.

